# How should policymakers, funders, and research teams mobilize to build the evidence base on universal early years services?

**DOI:** 10.1017/S1463423624000550

**Published:** 2024-12-10

**Authors:** Katie Harron, Sally Kendall, Catherine Bunting, Rebecca Cassidy, Julie Atkins, Amanda Clery, Eirini-Christina Saloniki, Francesca Cavallaro, Helen Bedford, Louise Mc Grath-Lone, Mengyun Liu, Jenny Woodman

**Affiliations:** 1 UCL Great Ormond Street Institute of Child Health, London, UK; 2 Centre for Health Services Studies, University of Kent, Canterbury, UK; 3 Care City Community Interest Company, London, UK; 4 Department of Applied Health Research (UCL) & NIHR Applied Research Collaboration North Thames, London, UK; 5 The Health Foundation, London, UK; 6 UCL Social Research Institute, London, UK

**Keywords:** early years, evaluation, evidence, Healthy Child Programme, health visiting, proportionate universalism, Public health service

## Abstract

Health visiting in England is a universal service that aims to promote the healthy development of children aged under five years and safeguard their welfare. We consulted stakeholders about their priorities for research into health visiting and also used these consultations and a literature review to generate a logic model. Parents wanted research to explore how health visiting teams can provide a caring, responsive, accessible service (the mechanisms of change). Policymakers, commissioners, and clinical service leads wanted descriptions and evaluations of currently implemented and ‘gold standard’ health visiting. The challenges to evaluating health visiting (data quality, defining the intervention, measuring appropriate outcomes, and estimating causal effects) mean that quasi-experimental studies that rely on administrative data will likely underestimate impact or even fail to detect impact where it exists. Prospective and experimental studies are needed to understand how health visiting influences infant–parent attachments, breastfeeding, childhood accidents, family nutrition, school readiness, and mental health and well-being.

## Background

The Healthy Child Programme (HCP) 0-5 is a universal service that aims to promote health and development and reduce inequalities among babies and preschool children in England. The HCP 0-5 is led by health visitors: specialist child and family public health nurses for children under five years of age who lead mixed skill teams, working with other community, public and specialist services (Public Health England, 2021). Local health visiting teams should offer five health reviews to every child and family between late pregnancy and the child’s third birthday, alongside access to community resources such as Family Hubs. These five mandated contacts provide an opportunity for health visiting teams to identify children and families in need of additional support from targeted and specialist services (including further health visiting contacts). The remit of health visiting is wide, including: health promotion and support with feeding and weaning, all aspects of child development, the home environment and family system, family health including maternal mental health and child welfare and safeguarding (in partnership with children’s social care). Alongside standard health visiting, some local areas also commission other nurse-led home-visiting programmes for families with young children such as Maternal Early Childhood Sustained Home-visiting (MECSH) program or the Family Nurse Partnership (FNP). Health visiting is an example of a service designed according to principles of ‘proportionate universalism’ which means that there is a universal service for all families, with extra support and service given according to a family’s need at any given time (Marmot *et al.*, 2010).

The HCP 0-5 faces significant organizational and structural challenges to effective implementation including: a shortage of qualified health visitors, budgets reduced by 19% in real terms since 2015 (Finch and Vriend, 2023) and high levels of disadvantage and need in young children. Just under 20% of children in England have below-expected development by age two, with high inequality (Cattan *et al.*, 2023). A third of young children in England live in poverty (Joseph Rowntree Foundation, 2024), and 41% of children have adversity coded in their own or their parents’ primary care record by age two (Syed *et al.*, 2023).

Research evidence on the practice and impact of health visiting can help local leaders design, commission, and deliver health visiting in the context of high need and scarce resource. Evidence can also be used to inform national policy on core elements of the universal health visiting offer and make a case for spending at both national and local level. Although the evidence base on health visiting is growing (Woodman *et al.*, 2022, Fraser *et al.*, 2022, Harron *et al.*, 2023, Barlow, 2023), many questions remain.

In autumn 2022, we consulted stakeholders and conducted a literature review as part of a larger study to evaluate the impact of health visiting on child and maternal health outcomes (Harron *et al.*, 2023) in order to identify priorities for research into health visiting and generate a logic model of health visiting. A logic model is a diagram explaining how the activities and qualities of health visiting might work to influence specific child and family outcomes (i.e. explaining Programme Theory). An important part of a logic model is articulating the theorized ‘mechanisms of change’ of an intervention, which are the necessary process or steps by which an activity or component of the intervention has the potential to bring about the desired change.

## Methods

We consulted with stakeholders to identify their priorities for research into health visiting. We conducted three workshops with parents of preschool children (nine mothers, five fathers) and four roundtables with policy colleagues from the Department of Health and Social Care in England (nine participants). In each group, we applied a modified nominal group technique and used voting to prioritize the research questions that had emerged during the discussion. We carried out five in-depth interviews with six key informants, comprising: two clinical leads of health visiting (trained health visitors now responsible for the day-to-day running of the health visiting service within their local area); two Local Authority commissioners of health visiting (Public Health Consultants); and a joint interview with two representatives from Institute of Health Visiting (a national professional membership charity advocating for and supporting high quality health visiting for families). The clinical leads and commissioners came from four different areas in England.

We synthesized the consultation results with learning from recent academic and policy literature to produce: (i) a set of questions to guide future research into health visiting; (ii) a logic model of health visiting (which builds on work in Scotland (Doi *et al.*, 2021)); and (iii) a discussion of methodological challenges.

## Results

Table 1 presents the health visiting research questions that were most important to parents and professionals. The priority areas span delivery, workforce, diverse family need, and improving data for intelligence.

**Table 1.** Stakeholder priorities for research into health visiting in England

Theme	Priority questions	Priority reported by		Study design needed	
		Parents	Professionals	Impact	Descriptive
Delivery	**1. Performance:** What does a gold standard health visiting service look like and how much does it cost?		✓	✓	✓
	**2. Integration:** How does health visiting work within the wider context of the Healthy Child Programme and with other services: GP, hospital, referrals, schools, preschools, and with Integrated Care Boards? Does health visiting work differently (and how/why?) when there are good community spaces, safe places, and community hubs? What is the impact of prioritized referrals from health visiting to other services, including dietitians, GP, and breastfeeding?	✓	✓	✓	✓
	**3. Innovation:** What are the innovations in delivery (including virtual contacts) that come from cost-efficiency pressures and what is their impact on family experience and outcomes?	✓	✓	✓	✓
	**4. Continuity of care:** How much does seeing the same/different staff members’ impact on family outcomes?	✓	✓	✓	
	**5. Providers:** What is the impact of different provider models, e.g. NHS, local authority, and social enterprise?		✓	✓	
	**6. Balance of intensive and universal provision:** How does delivering child protection work impact the ability of health visiting teams to deliver universal mandated contacts?		✓		✓
	**7. Relational care:** How can health visitors and other staff provide meaningful care (not just signposting or tickboxing) and build a non-judgemental relationship with parents?	✓			✓
	**8. Public perceptions:** How can we improve public understanding and expectations of health visiting?	✓	✓		✓
Workforce	**9. Employment conditions:** What conditions, skills, and training are needed for the health visiting workforce to deliver a good service?	✓	✓		✓
	**10. Skill mix:** What impact does it have if certain activities are delivered by health visitors, staff nurses, nursery nurses or other members of the health visiting team and how does this vary for different types of families? (Including medically trained staff versus other)	✓	✓	✓	
Response to diverse family need	**11. Universal service:** Can we evidence the value of the universal service?		✓	✓	
	**12. Targeted and specialist support:** Can we evidence the value of additional or opportunistic contact over and above the mandated universal contacts, including intensive work with high need families?		✓	✓	
	**13. Whole family approach:** What are the impacts of health visiting and the public health investment for the under 5s, not just for the child but across all family members?		✓	✓	
	**14. Geographical variation in family need**: How do the population needs of rural versus urban areas and North versus South areas of England influence the delivery and impact of health visiting?		✓	✓	✓
	**15. Transition to parenthood:** What do first-time parents need and how can they best be supported by the health visiting team?	✓			✓
Data	**16. Data quality:** What are the facilitators of local authorities collecting good quality data on health visiting activity and transferring that data to the national Community Services Dataset?		✓		
	**17. Workforce data:** What data do we need to understand how to train and support the workforce?		✓		

**Figure 1.**
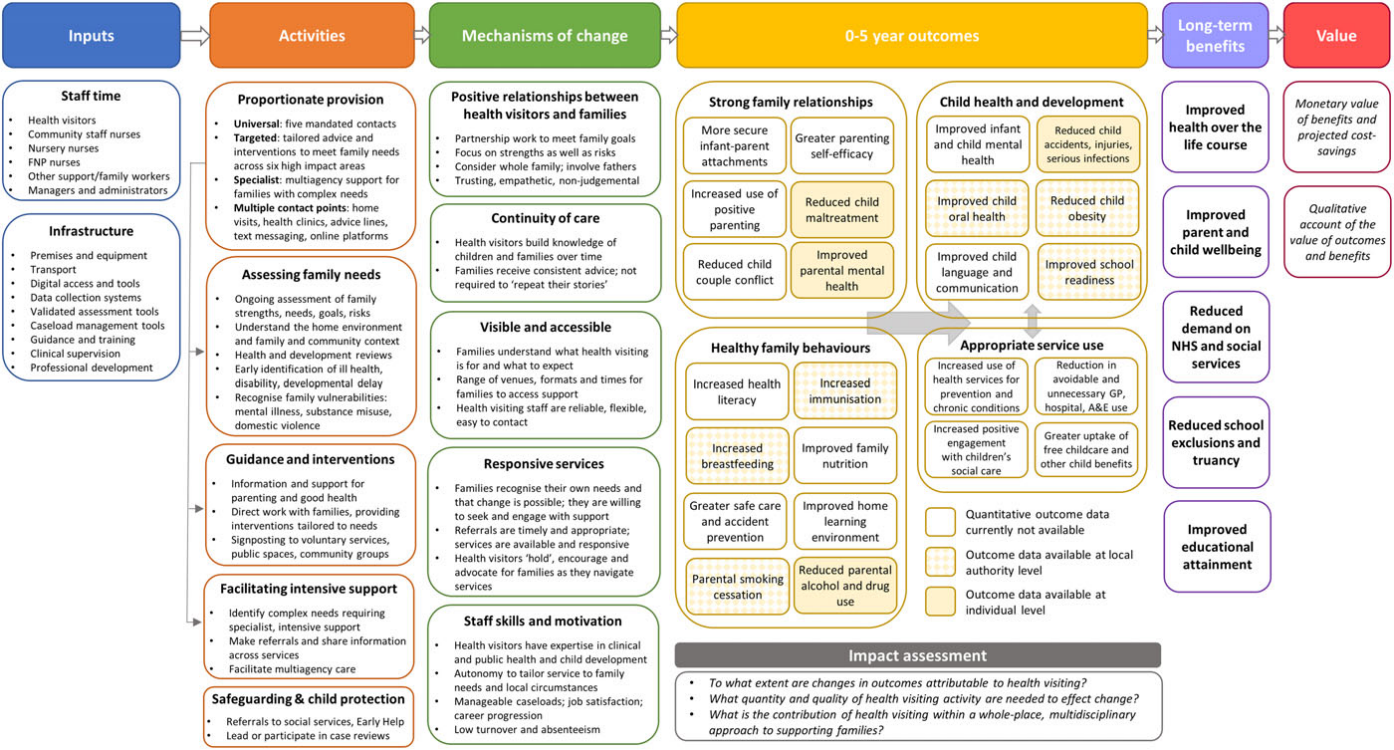
Logic model of health visiting in England.

Parents in our workshops expressed the need for a positive, non-judgemental relationship with their health visiting team, continuity of care, visible and accessible health visiting with a clearly articulated purpose, responsive services, and a motivated and skilled workforce. This is consistent with existing literature (Olander *et al.*, 2019, Cowley *et al.*, 2013). As our logic model (Figure 1) shows, these service characteristics can be seen as mechanisms of change, by which health visiting has the potential to influence child and family health (Cowley *et al.*, 2013).

Professionals emphasized the pressing need for evidence on the value of the universal element of the service and want to tease out the differential or cumulative impact of additional health visiting contacts and targeted support over and above the universal service. Professionals also identified priorities relating to implementation, the place of health visiting in the wider system, and innovation for cost efficiency.

In our logic model (Figure 1), we describe the potential impacts of health visiting that may be visible and measurable within the preschool period of a child’s life (‘0-5y outcomes’): strong family relationships, child health and development, healthy family behaviours, and appropriate service use. Figure 1 shows that we cannot use currently available national administrative data in England to measure these outcomes. These 0-5y outcomes have the potential to influence longer term outcomes such as improved parent and child well-being and reduced health service use (Figure 1) which can be monetized, enabling a comparison of the benefits and costs of the health visiting service.

Over half of the research priorities identified by stakeholders indicate a need for studies evaluating the impact of health visiting on child and family outcomes. However, generating evidence on the impact of health visiting presents significant methodological challenges and is underpinned by a need for descriptive research. We discuss these methodological challenges below.

## Discussion: challenges to evaluating health visiting, with recommendations

### Challenge 1: defining the intervention (in theory and in practice)

Health visiting delivery varies across populations, across geography, and over time. Any evaluation of health visiting will require a significant amount of descriptive work to understand how the service is being delivered in the community or period of interest.

Given the financial pressures that local authorities are experiencing, there may be a sizable difference between the theorized activities and mechanisms of change described in Figure 1 and the reality of ‘on the ground’ health visiting provision. One of our informants asked ‘What answer will you get if you evaluate a broken system?’, referencing a situation where (as others have described it) services are on their knees, mechanisms of change may be undermined and the service may not be able to achieve theorized impact (Wilkinson, 2022).

**Recommendation 1a:** There is a need to describe as well as evaluate health visiting as currently implemented including sub-optimal and cost-saving versions of services. This will include quantitative work using data produced by the health visiting service to analyse patterns of health visiting delivery and qualitative work in local areas to understand how services are organized and experienced on the ground.

**Recommendation 1b**: Policymakers, practitioners, and research teams should collaborate to describe and then implement and evaluate a ‘gold standard’ version of health visiting.

**Recommendation 1c:** Innovations and policy in health visiting should support and strengthen the theorized mechanisms of change outlined in our logic model. Evaluations should measure these mechanisms as well as child and family outcomes.

### Challenge 2: estimating causal effects

Health visiting is a long-standing universal intervention: there is no readily available group of families who do not receive health visiting to be used as a comparison group in the UK. This makes it difficult to estimate the causal effect of health visiting, i.e. the difference in the health outcomes of families who receive health visiting support and the health outcomes that those families would have experienced had they not received that support.

Instead, researchers can exploit the naturally occurring variation in organization and delivery of health visiting across England to compare outcomes in families that have received different types, timings, or intensities of the service. Some information on family characteristics, outcomes, and health visiting contacts can be extracted from routinely collected administrative datasets with whole-country coverage and cost-efficiency. However, there is a high risk of ‘confounding by indication’ because families with higher need who are at risk of poorer outcomes will receive more intensive health visiting support. Studies that fail to account for variation in family need may underestimate the effect of health visiting, or even find a spurious association between increased health visiting and poorer health outcomes. Quasi-experimental designs such as propensity score matching can minimize this form of bias by comparing the outcomes of families that are similar in terms of demographic, socio-economic, and health factors but receive different levels of health visiting support. However, capturing and accounting for all the relevant characteristics is problematic and residual confounding may remain (Cavallaro *et al.*, 2023).

**Recommendation 2a:** When comparing the outcomes of children who receive different levels of health visiting support, researchers will need to carefully identify and control for confounding factors at both family and system level.

**Recommendation 2b:** Policymakers, funders, and researchers should look to randomized study designs as a long-term goal, where promising or ‘gold standard’ models of health visiting are implemented in an experimental way at scale and outcomes are compared between families receiving and not receiving the ‘intervention model’ of health visiting. Experimental designs will be more expensive and difficult than quasi-experimental designs but will generate more robust evidence of the impact of different aspects of delivery.

### Challenge 3: measuring appropriate outcomes

Health visiting is theorized to influence the health and well-being of child and other family members across multiple domains of their lives (Figure 1). Advances in linking administrative health data with education and social care data across the United Kingdom (Mc Grath-Lone *et al.*, 2021) make it possible to investigate hospital attendance patterns and, potentially, ‘school readiness’ of children who received different levels of health visiting support. It remains challenging to measure benefits that accrue across the wider system using administrative data, such as improved home learning environment, parental health and well-being, or access to childcare (see Figure 1). If these benefits are detected, they may be difficult to attribute to health visiting alone.

Outcomes available in administrative data can be difficult to interpret. For example, emergency department attendance may reflect child safety issues relating to ingestions or injuries, but could also reflect appropriate healthcare seeking behaviour if a family cannot access services in the community (e.g. GPs). Increases in child protection plans could reflect an increase in child maltreatment but may also indicate improved access to children’s social care or better recognition by practitioners.

**Recommendation 3a:** Researchers need to be careful in their choice and interpretation of outcomes when evaluating health visiting.

**Recommendation 3b**: Prospective studies with primary data collection (observational or experimental designs) would allow researchers to measure mechanisms of change such as positive relationships between health visiting and families and continuity of care. These mechanisms of change have the potential to impact proximal (0-5y) outcomes (Kendall and Bloomfield, 2005), which can also be measured using primary data collection, including parent–child interaction, health literacy in the family, or home learning environment (see Figure 1). Linking administrative data to birth cohort studies might offer similar advantages to primary data collection (Hardelid *et al.*, 2021).

### Challenge 4: availability of data

The Office for Health Improvement and Disparities publishes aggregate data on health visiting activity by local authority, by quarter (Office for Health Improvement and Disparities, 2021). Many research questions require individual-level data, which is available in the Community Services Dataset (CSDS). The CSDS contains records of health visiting contacts including date, location, and duration of contact (Fraser *et al.*, 2020). Each local authority provider is responsible for submitting these data centrally to NHS England. However, completeness varies over time and across the country, and the dataset is described as experimental (NHS Digital, 2023). Key variables (such as the job role of the person delivering a contact) are often missing.

**Recommendation 4:** Policymakers, funders, and researchers can help to build the evidence base on early years services by supporting and encouraging the provision of accurate and comprehensive local data to CSDS.

## Conclusions

We found that parents want to know how health visiting teams can implement the caring, responsive service they value and which our logic model suggests is a necessary step to health visiting achieving positive outcomes for babies, young children, and families. Commissioners, service leads, and policymakers would like descriptions and evaluations of currently implemented and ‘gold standard’ services. Policymakers, funders, and research teams should mobilize to support research answering these questions.

The challenges to evaluating health visiting (data quality, defining the intervention, measuring appropriate outcomes, and estimating causal effects) mean that quasi-experimental studies that rely on administrative data will likely underestimate impact or even fail to detect impact where it exists.

In the short-term, in order to answer our stakeholder’s pressing questions, researchers should use administrative data to describe health visiting in all its variation. When administrative data and quasi-experimental methods are used to evaluate current practice, researchers should take into account the likely underestimate of impact. In the medium term, local areas can partner with research organizations to collect primary data through prospective or experimental studies to investigate the effect of health visiting as currently implemented on a wider range of outcomes, such as influences infant–parent attachments, breastfeeding, childhood accidents, family nutrition, school readiness, and mental health and well-being (as suggested by our logic model Figure 1). In the long term, policymakers, funders, researchers, and parents can work together to develop and evaluate innovations in health visiting delivery which can be tested using experimental study designs. Innovations in health visiting should strengthen the theorized mechanisms of change outlined in our logic model of health visiting.
